# Assessment of ventricular function after total cavo-pulmonary derivation in adult patients: Interest of global longitudinal strain

**DOI:** 10.34172/jcvtr.2023.32880

**Published:** 2023-12-30

**Authors:** Kaouther Hakim, Nouha Mekki, Rihab Benothmen, Mokbli Malek, Jarray Abdelkader, Msaad Hela, Habiba Mizouni, Ouarda Fatma

**Affiliations:** ^1^Pediatric Cardiology Department, La Rabta University Hospital of Tunis, Tunisia; ^2^Radiology Department, La Rabta University Hospital of Tunis, Tunisia

**Keywords:** Univentricular heart, Fontan operation, Echocardiography, Magnetic resonance imaging, Ventricular function, Global longitudinal strain

## Abstract

Ventricular dysfunction is the most frequent complication in adult patients post-Fontan completion. Through this work, we aim to evaluate ventricular systolic function by conventional echographic parameters and by global longitudinal strain (GLS) to determine the prediction of early ventricular systolic dysfunction. This is a prospective monocentric study enrolling 15 clinically stable adult Fontan patients with preserved ejection fraction (EF). Myocardial deformation study by GLS with speckle tracking technique in addition to a standard Doppler transthoracic echocardiography (TTE) was performed. Cardiac magnetic resonance imaging (CMR) was also performed. A comparison of echocardiographic and CMR parameters was made. In comparison to CMR-derived EF, we found a significant correlation with GLS and TTE-derived EF (*P*=0.003 and 0.014). We divided our population into two groups based on the cut-off value of 50% of CMR derived EF. Comparison of GLS in both groups showed a significant correlation (*P*=0.003). A cut-off value of -13.3% showed sensitivity of 67% and specificity of 100%. GLS has a moderate diagnostic value for systolic myocardial dysfunction in the population of adult patients with Fontan circulation.

## Introduction

 Total cavopulmonary connection (TCPC) is the final palliative staging procedure for anatomical single ventricles (ASV), in the presence of a unique atrioventricular connection as defined by Anderson and colleagues.^1^ The method was first introduced by Fontan in 1971.^2^ Since then, various surgical techniques have been proposed over the years. Despite considerable progress, long-term results remain compromised. Patients may be affected by ventricular dysfunction after TCPC. In fact, it is the main cause of morbimortality in this population.^3^

 Cardiac magnetic resonance imaging (CMR) represents the imaging technique of reference in the assessment of volumes and ejection fraction (EF) in the presence of univentricular anatomy. Its lack of availability and high cost are the main limitations of its routine use.

 Transthoracic echocardiography (TTE) is limited due to morphological heterogeneity and the complexity of the underlying congenital heart diseases. The validity of conventional echographic parameters remains controversial.

 The speckle-tracking technique for measuring global longitudinal strain (GLS) has been widely applied over the past two decades in the field of pediatric and congenital heart disease, including feasibility studies in single ventricles for the detection of subclinical ventricular dysfunction.^4,5^

 Our work aims to study the validation of conventional echographic parameters and GLS compared to CMR-derived EF in our population.

## Materials and methods

###  Patients

 We performed a prospective study enrolling 15 patients with ASV who underwent Fontan completion between 1995 and 2020 in our institution.

 Non-inclusion criteria were: patients on permanent atrial fibrillation or atrial flutter, and patients with significant valve regurgitation (greater than grade II).

 Inclusion criteria were: an underlying ASV, preserved TTE-derived EF (> 50%), minimum age of 18 years old, and minimal duration of follow-up post-Fontan completion of 2 years.

 Exclusion criteria were: limited echogenicity, contra-indications for CMR (patients with implanted ICDs and pacemakers), and patients with claustrophobia or mental retardation.

 Surgical and anatomical details were extracted from the corresponding medical files.

 A routine physical examination was performed for all patients. It included history taking to evaluate the degree of dyspnea on exertion (NYHA functional classification) and other associated symptoms. Measurements of height, weight, peripheral oxygen saturation, and blood pressure were performed.

 A standard 12-lead EKG to determine heart rhythm, heart rate, and QRS duration was also performed.

###  Echographic data

 All patients included in our study underwent detailed transthoracic echocardiography in our center. The studies were performed by a single investigator following a standardized protocol using the appropriate transducer of a GE VividT8 equipment. An electrocardiographic tracing was essential for identifying systole and diastole and for measuring GLS. A scale between 12 and 20cm/s was used.

 The following measurements were obtained from the average of at least three cardiac cycles.

####  Conventional echocardiographic parameters

 After an anatomical study, different conventional echocardiographic measurements were taken according to the latest recommendations of the American Society of Echocardiography (ASE) by assimilating the single ventricle to a left ventricle.

 Using the Simpson method, volumes and ejection fraction were measured by tracing manually endothelial contours of the dominant ventricle at the end-diastolic and end-systolic phases in apical 2-chamber and 4-chamber views.

 Pulsed wave tissue doppler was also used to measure peak annual systolic velocity (S’) and myocardial performance index (MPI). The corresponding measurements were obtained at the level of the basal segment of the lateral wall of the functionally atrioventricular valve in the setting of controlateral valve atresia. In the presence of two valves or a unique valve, the average of measurements at both walls of the dominant ventricle was obtained.

####  Speckle tracking acquisition and analysis

 The image frame rate was set at 60 frames/s. Recordings of 3 cardiac cycle loops were obtained in 2, 3, and 4-chamber apical views. In order to improve the quality of the tracking, we opted for a maximum depth as well as a minimum width of the acoustic window.

 For strain analysis, AFI software was used. Within the end-systolic phase, manual tracing of endocardial contours of the dominant ventricle was made. Based on the black-and-white transition, the software automatically generated epicardial contours. These borders can be retraced by the operator if judged necessary. The software then followed the myocardial deformation during the cardiac cycle according to its tracking algorithm. When strain curves correlated well with visual inspection, the tracking was approved. The ventricle is then automatically divided into 17 segments according to the model proposed by the ASE. The software generated the regional longitudinal strain of each segment to conclude the GLS which corresponds to the average of these measurements.

###  CMR data

 All patients underwent CMR in our center within a maximum of 2 months of the echocardiographic examination. The studies were performed following a standardized protocol with a GE 1,5 Tesla MRI scanner. In short, the protocol included multi-slice, multiphase, steady-state cine MR acquisitions in long-axis planes, and short-axis cine acquisitions from the base to the apex of the heart. Consequent acquisitions were obtained post Gadolinium injection.

 CMR data analysis was performed by a single observer using GE Medical Systems software. Automatic contouring of the endocardial and epicardial limits was rectified by the operator if necessary. Measurements of end-systolic and end-diastolic volumes (ESV and EDV respectively), mass, and ejection fraction of the dominant ventricle were obtained. Decreased single ventricle systolic function was defined as inferior to 50%.

 In addition, Late Gadolinium Enhancement (LGE) imaging, native and contrast-enhanced T1 mapping were performed to evaluate the presence and the extent of myocardial fibrosis.

 Moreover, angio-CMR and 4D flow sequences were also performed following the protocol to assess the permeability of the TCPC and the pulmonary branches, and to study the corresponding flow dynamics.

###  Statistical analysis

 Statistical analysis was performed using SPSS statistics 21.0. Quantitative data are presented as mean ± standard deviation. The comparison between two groups was performed using a two-tailed t-test. Correlation coefficients were calculated using Pearson’s or Spearman’s formula depending on data distribution. ROC curves were analyzed to determine the cut-off points corresponding to the highest values of sensibility and specificity. For all calculations, a *P* value < 0.05 was considered statistically significant.

## Results

###  Demographic data

 In our cohort, a masculine predominance was noted (9 patients). The average age was 33,6 ± 10,1 years (20-60 years), and the mean follow up-time since Fontan completion was 18,1 ± 8,6 years (Table 1).

**Table 1:** Patients’ characteristics.

**Parameters**	**Mean**	**Minimum**	**Maximum**
Age at Fontan completion (y)	18,1	5	39
Age at evaluation (y)	33,6	20	60
QRS duration (ms)	101,3	90	120
SpO2 (%)	94,3	88	98
BSA (m^2^)	1,73	1,42	2,19

BSA: body surface area, SpO2: peripheral oxygen saturation, y: years.

 The underlying congenital heart disease was tricuspid atresia in 6 patients, double inlet single ventricle in 6 patients, single ventricle with common atrioventricular valve in 2 patients, and mitral atresia in one patient. Left single ventricle morphology was predominant in 11 patients, and 4 patients had right or intermediate morphology of the dominant ventricle.

 Prior staging operations were performed on the majority of our patients (12 patients). Systemic-to-pulmonary shunts, pulmonary artery bandings, and partial cavo-pulmonary connections were performed in 9, 2, and 8 patients respectively. 10 patients underwent Fontan completion entailing an extracardiac conduit, 4 had intra-atrial lateral tunnels, and one had a classic Fontan operation. Only 3 TCPCs were fenestrated.

 3 patients underwent additional surgeries: 2 atrioventricular annuloplasties and a Damus Kaye intervention for subvalvular aortic stenosis in one case.

 6 patients developed arrhythmias in the course of follow-up. A recently diagnosed arterial hypertension and type 2 diabetes were noted in two patients.

###  Clinical and paraclinical data

 Most patients were asymptomatic at the time of their evaluation. Only 2 patients reported class II dyspnea on exertion.

 All patients were undergoing anticoagulant treatment. 4 were also on beta blockers for the control of associated arrhythmias.

 Blood pressure measurements were normal for all patients, except for one patient who had recently been diagnosed with arterial hypertension. The mean peripheral oxygen saturation was 94,3% (88-98%). All patients were on sinus rhythm. The mean QRS duration was 101,3 ms (90-120 ms).

Table 1 includes all descriptive statistics in this study to determine the mean, minimum and maximum of each parameter.

###  Echocardiographic data

 TTE-derived EF was preserved in all patients with a mean value of 57,6% (50-69%). GLS was increased with a mean value of -15,1 ± 2,6%.

 The mean values of end-diastolic and end-systolic volumes were 62,9 ml/m^2^ and 27,5 ml/m^2^ respectively. A large variation in ventricular volumes was noted.

 Peak annual systolic velocity was decreased with a mean value of 7,1 ± 1,8 cm/s. MPI was increased with a mean value of 0,6 ± 0,2 (Table 2).

**Table 2:** Echocardiographic and CMR parameters.

**Parameters**	**Mean**	**Maximum**	**Minimum**
TTE-derived EDV (ml/m^2^)	62,9	120	31,6
TTE-derived ESV (ml/m^2^)	27,5	54,6	8
TTE-derived EF (%)	57,6	69	50
S’ peak velocity (cm/s)	7,1	11	4
MPI	0,6	1,08	0,26
GLS (%)	-15,1	-11	-19,4
CMR-derived EF (%)	55,3	73	38
CMR-derived EDV (ml/m^2^)	94,6	190	45
CMR-derived ESV (ml/m^2^)	43,2	100	17

CMR: cardiac magnetic resonance imaging, EDV: end-diastolic volume, EF: ejection fraction, ESV: end-systolic volume, MPI: myocardial performance index, S’: peak annual systolic velocity, TTE: transthoracic echocardiogaphy.

###  Strain analysis

**Figure 1:**
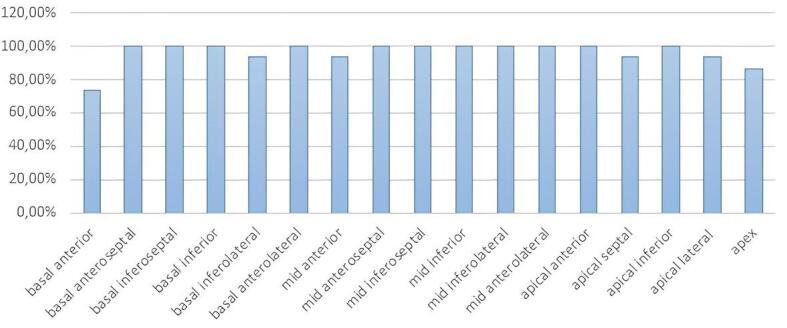
Percentage of segments visualized in TTE

 At least one myocardial segment was not qualified in 6 patients. Of a total of 255 segments analyzed, 4% were not visualized. The most concerned segments were the basal anterior and the apex (Figure 1). These findings were consequent to the limited acoustic windows and the particular anatomy of the ASV.

 The regional longitudinal strain was particularly increased in the basal anteroseptal, the basal anterior, and the mid-anterior segments.

 The pattern for most patients was increased regional longitudinal strain in the basal segments starting preferentially in the anterior and septal segments.

 In addition, heterogeneity in myocardial contraction was noted. In fact, the increase in regional strain in certain segments contrasts with the conservation of regional strain in adjacent segments, and a high prevalence of akinetic and dyskinetic segments (5,9%).

###  CMR data

 The mean CMR-derived EF was 55,3 ± 10,8%. 6 patients had ventricular systolic dysfunction (40%). The mean values of valumes and ventricular mass are represented in Table 2.

 CMR showed the absence of myocardial fibrosis based on LGE sequences. Interestingly, contrast-enhanced T1 mapping showed elevated values (for the cut-off value of 1050 ms) in different segments in 4 patients. This finding favors contraction heterogeneity previously noted in strain analysis.

 Angio-CMR and 4D flow sequences showed the permeability of the TCPC and the pulmonary branches for all patients.

Table 2 includes all descriptive statistics in this study to determine the mean, minimum and maximum of each echocardiographic and CMR parameter.

###  Correlation between conventional echocardiographic variables and GLS

 Conventional echocardiographic variables such as TTE-derived EF and Tissue doppler-derived parameters were statistically related to GLS. TTE-derived volumes did not correlate with GLS.

Table 3 describes the correlation between the conventional echocardiographic parameters and the GLS using Pearson corelation coefficient.

**Table 3:** Correlation between conventional echocardiographic parameters and GLS.

**Variables**	**R (Pearson)**	***P*** ** value**
TTE-derived EF	-0.54	0.036
TTE-derived EDV	0.23	0.39
TTE-derived ESV	0.34	0.20
S’ peak velocity	-0.75	0.001
MPI	0,67	0.006

CMR: cardiac magnetic resonance imaging, EDV: end-diastolic volume, EF: ejection fraction, ESV: end-systolic volume, MPI: myocardial performance index, S’: peak annual systolic velocity, TTE: transthoracic echocardiogaphy. *P*<0.05 is statistically significant.

###  Comparison between echocardiographic and CMR parameters

 We found no significant correlation between CMR-derived EF and Tissue doppler-derived parameters (*p* = 0.07 for S’ peak velocity, and *p* = 0,12 for MPI). Interestingly, a statistically positive correlation was found between CMR-derived EF, TTE-derived EF, and GLS (*p* = 0,014 and 0,003 respectively).

 In the same way, positive correlations were found between ventricular volumes of both imaging techniques (*p* = 0,02 and 0,005 respectively). However, there was an underestimation of end-diastolic and end-systolic volumes by TTE of 29% and 32% respectively.

 Patients were divided into two groups based on CMR-derived EF. Group 1 included patients with decreased EF and group 2 included patients with preserved EF.

 Analysis of echocardiographic parameters in both groups was performed. The comparative study was significant for TTE-derived EF and end-systolic volume. These parameters were significantly decreased in group 1. In the same way, the comparative study was significant for MPI and GLS. In fact, these parameters were significantly increased in group 1.

Table 4 describes the comparison of the echocardiogaphic parameters between grroups 1 and 2 using the independent Samples t-Test.

**Table 4:** Comparative study of echocardiographic parameters in groups 1 and 2.

**Variables**	**Groupe 1**	**Groupe 2**	***P*** ** value **
GLS	-12,9 ± 1,9	-16,5 ± 1,9	0.003
TTE-derived EF	54 ± 4,5	60 ± 4,9	0.032
MPI	0,76 ± 0,2	0,5 ± 0,16	0.015
S’ pic velocity	6,2 ± 1,7	7,7 ± 1,6	0.1
EDV	78 ± 16,7	52,8 ± 26,5	0.06
ESD	36,9 ± 12,8	21,3 ± 11,7	0.03

EDV: end-diastolic volume, EF: ejection fraction, ESV: end-systolic volume, GLS: global longitudinal strain, MPI: myocardial performance index, S’: peak annual systolic velocity, TTE: transthoracic echocardiogaphy. *P*<0.05 is statistically significant.

**Figure 2:**
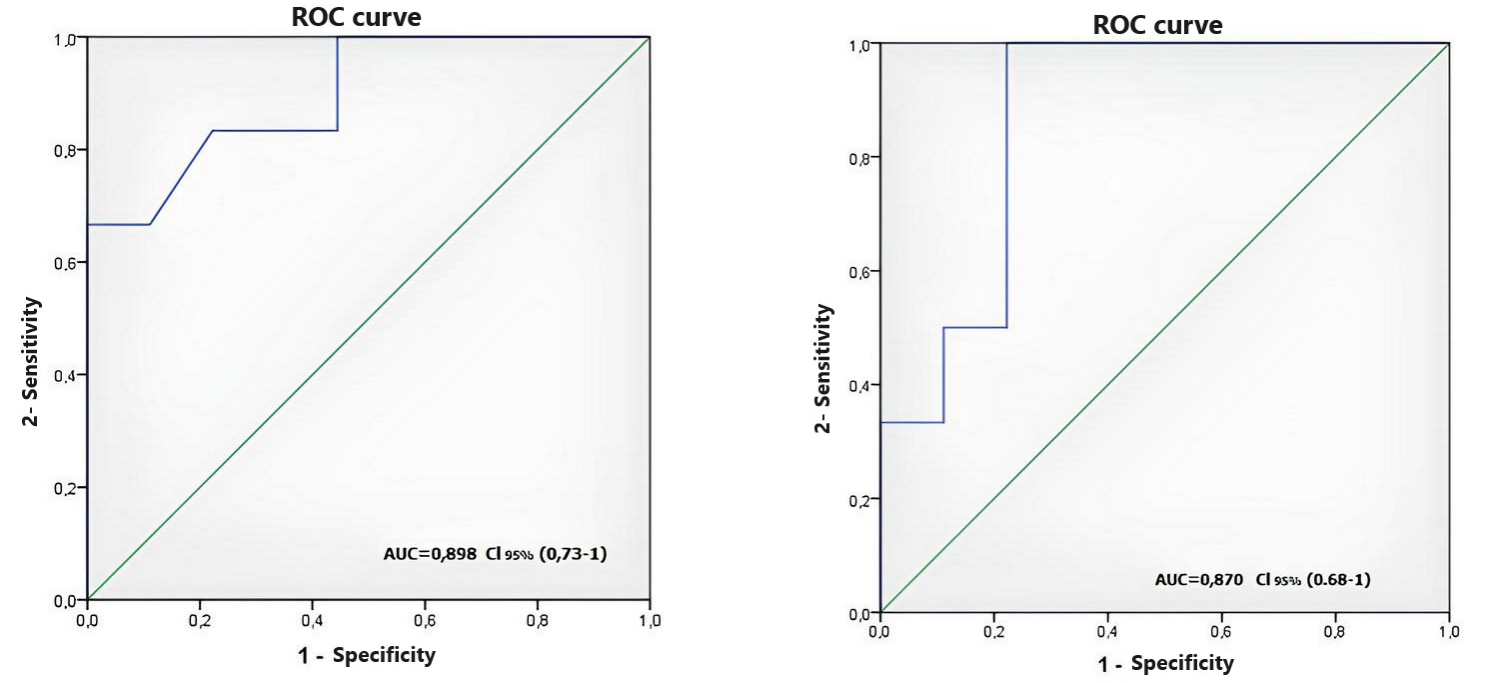
ROC curves of GLS and MPI. AUC = area under the curve, Cl = confidence interval

 After analyzing GLS and MPI independently, ROC curves and AUC were calculated for predicting a CMR-derived EF < 50%. Both curves have reached statistical significance (AUC = 0,898 and 0,870 respectively) (Figure 2).

 A cut-off point of 0,6 for MPI yielded 67% sensitivity and 78% specificity, and a cut-off point of -13,3%, for GLS yielded 67% sensitivity and 100% specificity.

 ROC curve analysis for TTE-derived EF and S’ peak velocity showed curves below the diagonal for both parameters.

###  Effect of the ventricle’s morphology

 When comparing the patients based on the dominant ventricle’s morphology, GLS and CMR-derived EF are statistically significant

Table 5 describes the comparison of echocardiographic and CMR parameters based on the morphology of the dominant ventricle using Pearson correlation coefficient.

**Table 5:** Comparison of echocardiographic and CMR parameters based on the morphology of the dominant ventricle.

**Variables**	**R (Pearson)**	***P*** ** value**
TTE-derived EF	0.91	0.08
S’ peak velocity	0.42	0.07
MPI	0.62	0.19
GLS	0.46	0.005
CMR-derived EF	0.07	0.002
CMR-derived EDVi	0.4	0.87
CMR-derived ESVi	0.27	0.33

CMR: cardiac magnetic resonance imaging, EDVi: indexed end-diastolic volume, EF: ejection fraction, ESVi: indexed end-systolic volume, GLS: global longitudinal strain, MPI: myocardial performance index, S’: peak annual systolic velocity, TTE: transthoracic echocardiogaphy. *P*<0.05 is significant.

###  Reproducibility

 The assessment of intra-observer reproducibility was performed by analyzing intra-class correlations of the different measurements of each parameter. The intra-rater agreements were good for GLS and S’ peak velocity and moderate for MPI and TEE-derived EF (Table 6).

**Table 6:** Intra-class correlation coefficient for different parameters.

**Variable**	**Intra-rater ICC**
TTE-derived EF	0.74 (0.5, 0.89)
S’ peak velocity	0.89 (0.77, 0.96)
MPI	0.75 (0.51, 0.89)
GLS	0.85 (0.69, 0.94)

EF: ejection fraction, GLS: global longitudinal strain, MPI: myocardial performance index, S’: peak annual systolic velocity.

## Discussion

 Most of the reported studies in the literature investigated the interest in Speckle tracking (ST) technique in the pediatric population.^4,6,7^ The current study is one of few to investigate the value of myocardial deformation imaging in comparison to CMR in adult patients with ASV post-Fontan completion. It demonstrates that GLS has a moderate diagnostic value in the detection of early ventricular systolic dysfunction in this population.

 It is well known that in the setting of Fontan physiology, changes in loading conditions lead to altered geometrical structure in the ventricle. Gewiling et al demonstrated that increased cardiac output and higher afterload are associated with ventricular dysfunction.^8^ This is mainly due to ventricular hypertrophy and increased sphericity.^9^ These structural changes leading to uncoordinated ventricular relaxation explain the high prevalence of ventricular dysfunction. In fact, decreased systolic function and reduced global longitudinal strain were previously reported independently from age, time to Fontan completion, and follow-up time since surgery.^9,10^

 In our study, segmental longitudinal strain analysis showed heterogenous contractility with evidence of a high prevalence of severely hypokinetic and akinetic segments adjacent to normokinetic segments. This finding was also established by CMR following a contrast-enhanced T1 mapping study.

 Recent studies have confirmed heterogeneity in regional function in accordance with our study.^9,11^ Factors like myocardial fiber orientation, fibrous tissue, and conduction delays have been associated with a higher prevalence of regional reduced deformation.^9,12^

 Correlations between GLS and conventional echocardiographic parameters were established in our series in accordance with the published study by Cho et al in adults with preserved EF and ventricular dysfunction by ischemic disease.^13^

 No statistically significant correlation was found between CMR-derived EF and Tissue doppler-derived parameters. These findings were previously described by various authors and suggest that these parameters are not sensitive for the detection of early ventricular dysfunction.^6,14^

 Although a positive correlation was found between CMR-derived EF and TTE-derived EF, ROC curve analysis showed a curve below the diagonal concluding to poor sensitivity and specificity.

 Not surprisingly, TTE-derived end-diastolic and end-systolic volumes were underestimated by 29% and 32% respectively. This was also demonstrated by previous studies and explained by the complex anatomy of the univentricular heart.^6,10^

 Despite the particular morphology of the dominant ventricle, the prevalence of excluded myocardial segments of the ST strain analysis is relatively low in all published series.^9,15,16^ The prevalence in our population was 4%.

 In recently published studies, myocardial deformation study using the ST technique is proven to be reproductive and faisable.^4,17^ Rios et al conducted a study including 100 patients, they concluded that this technique increased time acquisition by only 10.8 ± 5.5 minutes and reported excellent inter-observer reliability.^17^

 In our study, we demonstrated that GLS had the most significant correlation to CMR-derived EF (*P* = 0.003). In addition, it had better thresholds of sensibility and specificity (67% and 100% respectively) for a cut-off point of -13,3% compared to conventional echocardiographic parameters.

 Similarly, a study by Kaka et al demonstrated an increased GLS in the majority of patients even in the presence of preserved TTE-derived EF and normal E/e’ ratio, suggesting that abnormal GLS can serve as a precursor to decreased systolic and diastolic cardiac function.^18^

 Singh et al studied the accuracy and reproducibility of global strains measured by ST compared to CMR-measured values in 12 pediatric patients with tricuspid atresia. The authors concluded that both imaging techniques demonstrated close agreements for all myocardial segments, for both longitudinal and circumferential strain (*P* < 0.001 for both).^4^

 However, another study by Schmidt et al conducted on 15 adult patients concluded that only a moderate correlation was found between CMR-based Feature tracking and TTE-ST for longitudinal strain (coefficient of variability of 29.9%).^15^

 These contradictory results are possibly explained by the limitation of TTE in the obtention of reliable acquisitions in the presence of both complex anatomy and limited acoustic windows, particularly in adult patients.

 We found a significantly decreased CMR-derived EF in patients with left morphology compared to those with non-left morphology (*P* = 0.002). Interestingly, a significantly increased GLS was also found between both groups (*P* = 0.005).

 Although left ventricle morphology is theoretically more adapted to systemic pressures, the long-term effects of ventricle morphology are controversial.^16,19^ However, recently published studies demonstrate that based on CMR data, patients with non-left ventricle morphology are likely to have decreased systolic EF compared to those with left ventricle morphology due to the difference in myocardial fiber architecture.^12,20^

## Limitations

 Our study is mainly limited by the modest study sample size. This is particularly important in the study of myocardial deformation. A larger multicentric study including a larger population of patients covering the whole spectrum of the disease and especially including a larger number of patients with a morphologically right ventricle is necessary to confirm the results.

 Another possible limitation is the relative heterogeneity of patients’ demographic data. However, because the aim of this study was to examine the value of GLS and conventional echocardiographic parameters, we thought that these data would not influence study results.

 Finally, regarding the morphology of the dominant ventricle, the investigator could not be blinded for obvious reasons.

## Conclusion

 GLS-derived by the ST technique shows a significant correlation with CMR-derived EF. Since ST parameters are superior to conventional echocardiographic parameters in detecting early myocardial dysfunction, these results may improve the quantitative assessment of ventricular function. Unlike CMR, the study of myocardial deformation can be performed in routine practice. Until the advent of larger studies, routine clinical use of the ST technique cannot be advocated, but it should be considered as an adjunct to conventional echocardiographic parameters in the presence of Fontan physiology.

## Acknowledgments

 We would like to thank all patients included in this study for approval to participate.

## Competing Interests

 All authors declare no competing interests.

## Declaration of Interests

 All authors declare that they have no known competing financial interests or personal relationships that could have appeared to influence the work reported in this paper.

## Ethical Approval

 Additional informed consent was obtained from all patients.

## Funding

 Not applicable.
